# In vitro effects of imatinib mesylate on radiosensitivity and chemosensitivity of breast cancer cells

**DOI:** 10.1186/1471-2407-10-412

**Published:** 2010-08-09

**Authors:** Marion T Weigel, Linda Dahmke, Christian Schem, Dirk O Bauerschlag, Katrin Weber, Peter Niehoff, Maret Bauer, Alexander Strauss, Walter Jonat, Nicolai Maass, Christoph Mundhenke

**Affiliations:** 1 Department of Obstetrics and Gynecology, Breast Center, University of Kiel, Arnold-Heller Strasse 3, 24105 Kiel, Germany; 2 Department of Obstetrics and Gynecology, Breast Center, University of Aachen, Germany; 3 Department of Radiotherapy (Radiooncology), University of Kiel, Germany

## Abstract

**Background:**

Breast cancer treatment is based on a combination of adjuvant chemotherapy followed by radiotherapy effecting intracellular signal transduction. With the tyrosine kinase inhibitors new targeted drugs are available. Imatinib mesylate is a selective inhibitor of bcr-abl, PRGFR alpha, beta and c-kit. The purpose of this study was to determine whether Imatinib has an influence on the effectiveness of radiotherapy in breast cancer cell lines and if a combination of imatinib with standard chemotherapy could lead to increased cytoreduction.

**Methods:**

Colony-forming tests of MCF 7 and MDA MB 231 were used to study differences in cell proliferation under incubation with imatinib and radiation. Changes in expression and phosphorylation of target receptors were detected using western blot. Cell proliferation, migration and apoptosis assays were performed combining imatinib with doxorubicin.

**Results:**

The combination of imatinib and radiotherapy showed a significantly stronger inhibition of cell proliferation compared to single radiotherapy. Differences in PDGFR expression could not be detected, but receptor phosphorylation was significantly inhibited when treated with imatinib. Combination of imatinib with standard chemotherapy lead to an additive effect on cell growth inhibition compared to single treatment.

**Conclusions:**

Imatinib treatment combined with radiotherapy leads in breast cancer cell lines to a significant benefit which might be influenced through inhibition of PDGFR phosphorylation. Combining imatinib with chemotherapy enhances cytoreductive effects. Further in vivo studies are needed to evaluate the benefit of Imatinib in combination with radiotherapy and chemotherapy on the treatment of breast cancer.

## Background

Breast cancer is the most common malignant tumour in women. Albeit further increases in incidence, breast cancer related mortality has been reduced by screening and early detection programs, as well as optimized therapeutic options. Besides surgery, chemotherapy and radiotherapy, targeted therapies including endocrine, small molecule and antibody related therapies have been able to improve patient outcome [1-5].

In early and advanced breast cancer, radiotherapy is a common part of standard therapies. External beam radiotherapy (50 Gy, fraction dose 1.8 - 2 Gy, delivered over 30 - 35 days) is used for chest wall and total breast irradiation [6,7].

Radiotherapy targets intracellular DNA and causes strand breaks. The ability of tumour cells to repair radiotherapy modulated DNA breaks is limited. Unrepaired DNA breaks commonly lead to apoptosis, necrosis, cell cycle arrest or mitotic inactivity. Radio sensitivity depends on intrinsic factors, defined by genetic determination as well as on extrinsic factors like growth receptor signalling and their chemical or biological modulations [8].

Membrane tyrosine kinases play a key role in cell signalling. Aberrant expression or activation has an impact on breast cancer oncogenesis and progression. Tyrosine kinase inhibitors show activities against one or multiple targets and are able to inhibit tumour proliferation and in some cases angiogenesis at the same time [9].

Imatinib mesylate (Glivec^®^) was originally developed for tailored inhibition of the oncoprotein bcr-abl in chronic myeloid leukaemia (CML) and is today part of CML standard therapies. Besides abl and bcr-abl, imatinib also inhibits the activation of PDGFR α, β and c-kit and is currently used in research and treatment of solid tumours [10-13].

Cell lines from different solid tumours with c-kit and/or PDGFR expression have been tested previously for their response to imatinib. Cell growth of c-kit expressing cell lines of colon and small cell lung cancer could be inhibited in vitro and in vivo [14]. PDGFR activation occurs via an autocrine pathway or by ligands. Activation of PDGFR ß enhances chemotaxis, while cell motility is decreased after PDGFR α activation. Inhibition of both subtypes leads to apoptosis. Co-expression of PDGFR ß and stimulating ligands can be seen in many malignant lesions [15-19]. Immunohistochemistry revealed a broad PDGFR expression in breast cancer [20,21]. Increased expression of PDGF receptors correlates with an increased risk of distant metastasis, decreased response to chemotherapy and reduced overall survival [22,23]. In murine breast tumours inhibition of activated PDGFR ß by imatinib leads to reduction in tumour cell growth [20].

Imatinib has also an antiangiogenic effect and leads to apoptosis in tumour and endothelial cells by blocking PDGF-B signalling pathways [24-28]. In highly angiogenic glioblastomas imatinib showed radiosensitizing activity. Additionally an imatinib related inhibition of PDGFR β activation leads to a decrease in the interstitial pressure of solid tumours. This effect promotes an intracellular up-take of substances like cytotoxic agents [29,30]. Therefore it was suggested to introduce imatinib into clinical research and therapies of solid tumours like breast cancer expressing the specific cellular targets. Expression patterns of tyrosine kinases, relevant for imatinib action, have been described in human breast cancer cell lines previously. It is likely, that imatinib action in solid tumours is related to PDGFR β inhibition [31]. Our hypothesis was that imatinib could contribute to breast cancer therapy by serving as a potential chemo- and radiosensitizer.

## Methods

### Cell Culture

Human breast cancer cell lines (MCF 7 and MDA MB 231) were obtained from American Type Culture Collection, Rockville, USA. Cells were grown in RPMI 1640 medium containing 10% FBS and 1% penicillin/streptomycin at 37°C and 5% CO_2_. Their tyrosine kinase receptor and corresponding ligand expression patterns have been described previously [31].

### Ligand dependent proliferation and migration

Cells for proliferation assays were seeded at a density of 10,000 cells/ml on a 96-well plate. After an attaching period of 24 hours, medium was exchanged for medium containing only 1% FCS to reduce effects of included growth factors. Cell lines were incubated either with PDGF BB (R&D Systems, Minneapolis, MN, USA) (10, 25, 50, 75 or 100 ng/ml) alone or in combination with imatinib mesylate (IC50 6 μM, defined previously[31,32]) (generously provided by Novartis, Basel, Switzerland) which was added before application of PDGF BB. Medium was replaced every 48 hours. After six days of incubation, experiments were terminated using Cell Titer Aqueous One Solution Reagent (Promega) and cell proliferation was measured. For migration assays silicon spacers were inserted into 12-well plates before cells were seeded. At the point of cell confluency, spacers were removed and distances between migration fronts were measured. Cells were incubated with imatinib, PDGF BB (25 ng/ml) or their combination for 72 hours and cell migration was measured every 24 hours using Axio vision software (Zeiss, Germany). Experiments were set up in triplicates and performed three times to confirm results. For statistical analysis student's t-test was carried out and p-values < 0.05 were declared significant.

### Cell proliferation combining imatinib mesylate and doxorubicin

Cells were plated on 12-well plates and were allowed to attach for 24 hours. Then, cells were incubated with doxorubicin (Sigma-Aldrich, St Louis, MO, USA) in concentrations of 0.25 nM - 12.5 nM and imatinib (1.5 - 12 μM) alone and in combinations. Replacement of medium was carried out every 48 hours and cell proliferation was determined by trypsinization, trypan blue staining and cell counting after six days. Experiments were set in triplicates and repeated to confirm results. Drug interaction was assessed using the combinatory index, where CI < 1, CI = 1 and CI > 1 indicate synergistic, additive and antagonistic effects. Data analysis was carried out using the Calcusyn Software (Biosoft, Oxford, UK)[32,33].

### Apoptosis analysis

Breast cancer cell lines were seeded on chamber slides (MDA MB 231 20,000 cells/ml, MCF 7 40,000 cells/ml) and after attachment over night, cells were incubated with imatinib (IC50 6 μM), doxorubicin (1 nM, 2,5 nM and 5 nM) or the combination of both drugs for 24 and 48 hours. Slides were washed with PBS, cells were fixed with 4% paraformaldehyde and TUNEL assay was carried out according to manufacturer's protocol. Apoptotic cells were counted in five random fields using fluorescence microscopy and expressed as percentage of total cell number. Student's t-test was used for statistical analyses and p-values < 0.05 were declared significant.

### Radiation in vitro

Cells were cultured in T 75 cell culture flasks and seeded at a density of 4,000 (MDA MB 231) and 10,000 (MCF 7) cells/ml. After 24 hours of attaching, cells were incubated with or without imatinib in concentrations of 4 or 6 μM. Radiation was performed after another 24 hours using a telecobalt source (Theatron 780C, Phillips) emitting gamma radiation produced by cobalt 60 with energy of 1.33 MeV. In previous experiments a difference between radiation using cobalt 60 or iridium 192 could not be detected (data not shown). During radiation and transport, cells were stored in a specially designed isolated box guaranteeing a constant temperature of 37°C. A total dose of 10 Gy was applied fractionated in doses of 2 Gy per day on 5 consecutive days. Cells were counted using the trypan blue method 24 hours after each fraction. All experimental points were set-up in triplicates and repeated at least twice to confirm results.

### Colony forming test

Colony forming tests were carried out to detect the effect of radiation on cell vitality. 1 g of bactoagar (Becton, Dickinson & Co., Sparks, MD, USA) was boiled in 20 ml pure water and suspended with 20 ml culture medium. Separately 1,000 MDA MB 231 cells/ml and 3,000 MCF 7 cells/ml were added at 37.5°C. Different set-ups were used for each dose level. After polymerization the agar was covered with 2 ml of cell culture medium. Medium changes took place every seven days and after 10 (MDA MB 231) and 21 (MCF 7) days of incubation. Colonies consisting of more than 30 cells were counted. Plating efficiency was calculated by multiplying the number of colonies by 100 and dividing it by the number of cells plated. To determine the surviving fraction the number of colonies of treated cells was divided by the number of colonies of non radiated control cells. Statistical analysis was performed using the student's t-test and p-values < 0.05 were declared significant.

### Immunoblotting

To detect the influence of radiation and imatinib co-treatment on receptor activation, cells were lysed in lysis buffer (62.5 mM Tris-HCL (pH 6.8), 2% SDS, 50 mM DTT and 10% glycerol) and proteinase inhibitor was added. Protein determination was carried out using the Bradford method (Bio-Rad, Hercules, CA, USA). 30 μg of total protein were separated depending on molecular weight in SDS-PAGE. After transfer onto PVDF-membranes, membranes were blocked and incubated with primary antibody. Primary antibodies used were rabbit polyclonal anti-PDGFR β (Santa Cruz Biotechnology, Santa Cruz, CA), rabbit polyclonal anti-phospho-PDGFR β (Cell Signalling Technology, Danvers, MO, USA) and anti-β-actin (Sigma-Aldrich). After incubation with corresponding secondary horseradish peroxidase-conjugated antibodies, blots were developed using the ECL-system (Amersham Biosiences, Piscataway, NJ, USA).

Research reported has been carried out with the agreement of the ethics committee of the University of Kiel, Germany (AZ D 426/10).

## Results

In previous experiments we could determine that the cell lines used express receptors which are known imatinib targets. All cell lines express PDGFR β and abl. MCF 7 cells are positive for c-kit and PDGFR α is expressed by MDA MB 231. Despite their different expression patterns, imatinib has an anti-proliferative effect on both breast cancer cell lines with an IC 50 concentration of 6 μM [31,34].

### Imatinib inhibits PDGF BB dependent cell proliferation and migration

**Figure 1:**
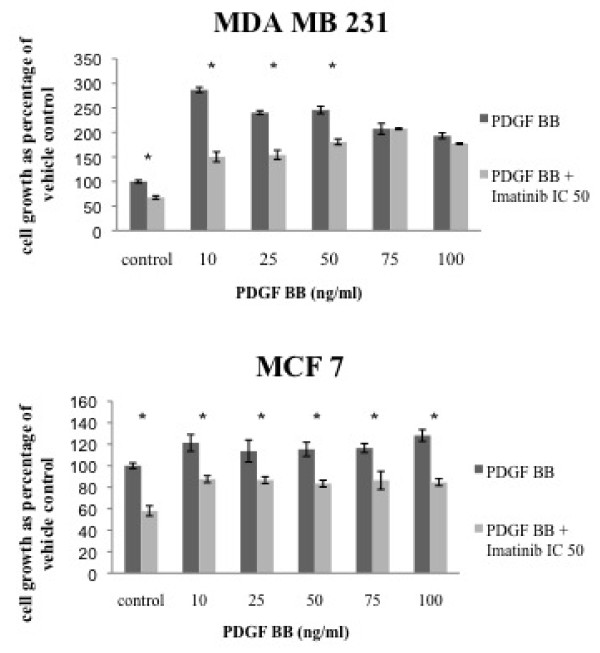
**Imatinib inhibits PDGF BB dependent cell proliferation in breast cancer cell lines**. Cells were incubated with increasing concentrations of PDGF BB and imatinib (IC 50). Cell growth was measured by MTT-assay.

In MDA MB 231 cells stimulation with PDGFR β specific ligand PDGF BB in concentrations of 10 ng/ml leads to an increased cell growth of 186 percent. Higher ligand concentrations are not able to induce incremental cell proliferation (Figure 1). On the other hand imatinib is able to reduce the proliferative effect of PDGF BB. MCF 7 cells react differently on growth factor application. PDGF BB induces a cell growth increase of about 20% among all concentrations used and imatinib is able to reduce cell proliferation by 50%. The cell growth factor PDGF BB increases cell growth in breast cancer cell lines. Response rates to ligand specific signal-transduction vary, but imatinib is able to exert its anti-proliferative effect on all breast cancer cells in spite of PDGF BB stimulation.

**Figure 2:**
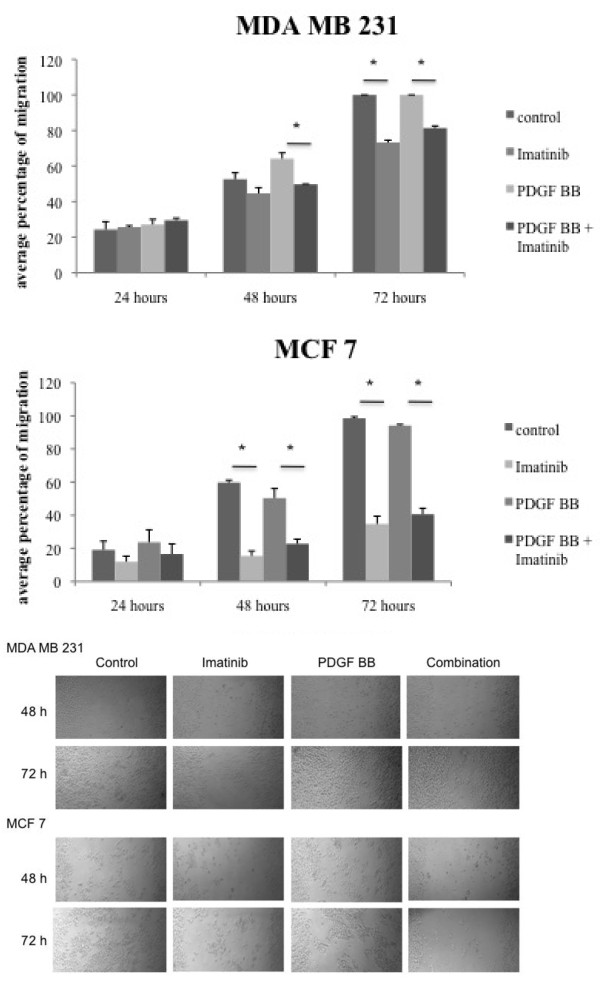
**Imatinib inhibits PDGF BB dependent cell migration in breast cancer cells**. Migration assays were performed in breast cancer cell lines. Cells were incubated with either imatinib, PDGF BB or their combination for up to 72 hours. Cell migration was measured and student's t-test was performed. * indicates p-values < 0.05. Representative images of cell migration after 48 and 72 hours of incubation are displayed.

MDA MB 231 cells show an increase in cell migration compared to the control upon stimulation with PDGF BB (Figure 2). Imatinib has the ability to inhibit cell migration in the absence of PDGF BB and this effect is also apparent in the presence of growth factor stimulation. In contrast, PDGF BB does not significantly alter cell migration of MCF 7 breast cancer cells, but imatinib reduces their migration significantly at 48 hours and as well at 72 hours of incubation. The tyrosine kinase inhibitor imatinib reduces cell migration of the studied breast cancer cell lines significantly and this anti-migratory effect persists in the presence of PDGF BB stimulation.

### Combination of imatinib and doxorubicin leads to enhanced cell growth inhibition

**Figure 3:**
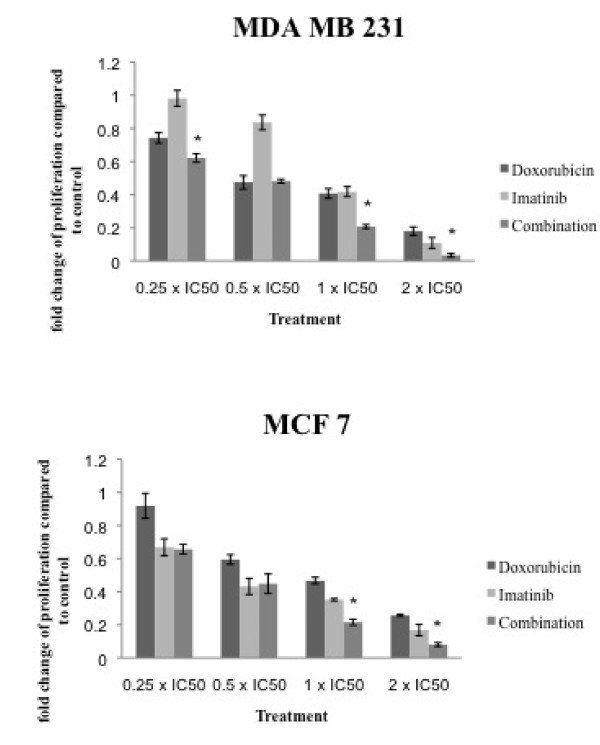
**Combination of imatinib with doxorubicin leads to enhanced cell growth inhibition**. Cells were incubated with doxorubicin or imatinib alone or in combination. Cell proliferation was assessed after six days of incubation. Combination indices were calculated using Calcusyn software. * = p-value 0.05 determined by student's t-test.

This experiment was set-up to detect if imatinib might be a useful add-on to the cytotoxic drug doxorubicin which is used in different therapeutic settings in breast cancer treatment. Therefore cells were incubated with increasing concentrations of doxorubicin and imatinib alone or in combination (Figure 3). Dose ratios were applied and combination indices were calculated (Table 1). An additive effect on cell proliferation can be detected in MDA MB 231 cells using a dose ratio of 1:6,000 (doxorubicin:imatinib), which contains the IC50 of both drugs. Among MCF 7 cells the combination reaches an additive effect in higher dose ranges, whereas the combination of doses lower than the IC50 shows an antagonistic effect. Both therapeutic drugs show an additive effect on cell growth inhibition when combined, which is pronounced in the triple negative MDA MB 231 cell line.

**Table 1:** Combination indices calculated for imatinib and doxorubicin co-incubations in breast cancer cell lines

MDA MB 231						
**Drug**	**Combination Index Values at**			**Dm (nM)**	**m**	**r**
	**ED 50**	**ED 75**	**ED 90**			

**Combination (1:6000)**	1.11214	1.01224	0.99765	0.39732	1.84658	0.96979
**Doxorubicin**	N/A	N/A	N/A	0.57999	1.15437	0.97662
**Imatinib**	N/A	N/A	N/A	5581.5821	2.92342	0.99784

						
**MCF 7**						

**Drug**	**Combination Index Values at**			**Dm (nM)**	**m**	**r**
	**ED 50**	**ED 75**	**ED 90**			

**Combination (1:1200)**	1.32605	1.10981	0.95853	2.03607	1.48647	0.99769
**Doxorubicin**	N/A	N/A	N/A	4.56443	1.58527	0.96108
**Imatinib**	N/A	N/A	N/A	7487.20892	1.04304	0.98481

ED 50 (effect dosis 50%), Dm (IC50) calculated, m (measures of sigmoidicity), r (correlation coefficient)

### Imatinib's effect on cell apoptosis

**Figure 4:**
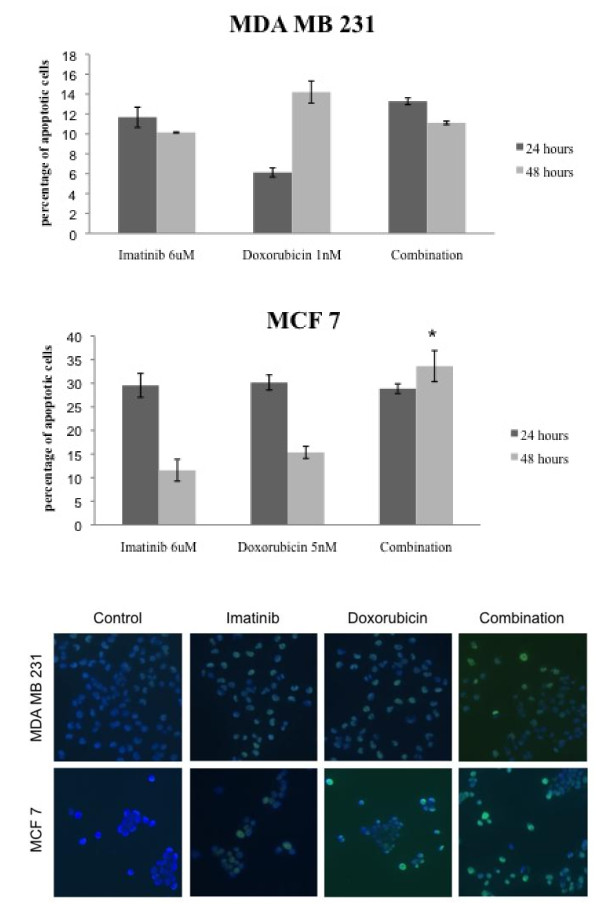
**Effect of imatinib and doxorubicin on apoptosis of breast cancer cells**. Cells were incubated with imatinib, doxorubicin alone or their combination for 24 and 48 hours. Apoptotic fraction was estimated by TUNEL assay and apoptotic cells were counted using fluorescence microscopy. For each sample, a total of five times 200-500 cells were scored and apoptotic fractions are expressed as percentage of total cells counted. Apoptotic cells show a green nuclear staining compared to vital cells.

To determine whether the combination of imatinib with doxorubicin leads to increased levels of cells undergoing apoptosis, TUNEL assays were carried out. Incubation of MDA MB 231 cells with imatinib induces apoptosis in 12 and 10% of cells after 24 and 48 hours. Cells incubated with doxorubicin over the same time show an apoptotic rate of 5% and 14%. The combination of both drugs does not increase the level of cells undergoing apoptosis at these specific time points (Figure 4). In contrast, apoptotic rates in the hormone receptor positive MCF 7 cells rise to comparable levels after 24 hours of incubation with either drug alone or their combination. Moreover, after 48 hours the amount of apoptotic cells decreases in imatinib and doxorubicin treated cells (11% and 15%), whereas the combination shows an increase in apoptotic cells to 34%. Imatinib and doxorubicin lead to apoptosis induction in both cell lines, but only in MCF 7 cells a further increase in apoptosis can be detected when drugs are combined.

### Effects of radiation on breast cancer cells

**Figure 5:**
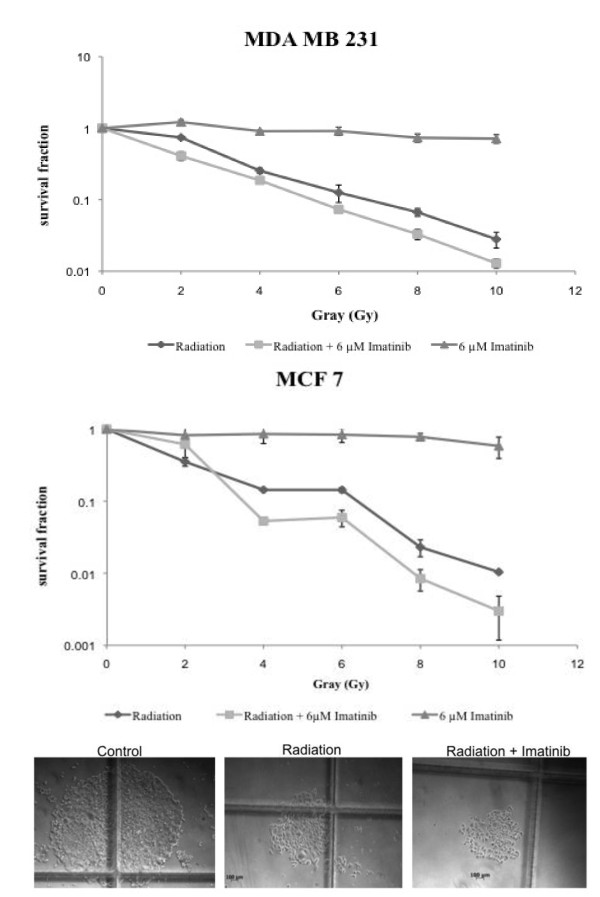
**Combination of imatinib with irradiation reduces breast cancer cell growth in vitro**. Cells were incubated with imatinib alone, received irradiation of 2 Gy per day for 5 days or the combination of both. Colony-forming tests were performed and the surviving fraction was calculated dividing the number of colonies of treated cells by the number of colonies of non radiated control cells. Representative colonies of MCF 7 cells are shown.

To detect the effect of irradiation on breast cancer cell lines, the estrogen receptor (ER) positive cell line MCF 7 and the ER negative cell line MDA MB 231 were selected. Fractionated radiation with 2 Gy per day on 5 following days to reach a total dose of 10 Gy was performed. The surviving fraction of each dose level was determined. Both cell lines show a decrease in the surviving fraction which correlates with the increasing dose levels (Figure 5). The decrease is stronger in the highly proliferative MDA MB 231 cell line.

### Combination of imatinib with irradiation reduces breast cancer cell growth

To evaluate if imatinib could enhance the effect of radiation on breast cancer cells, cells received either single radiation, imatinib incubation (IC50) or the combination of both. The surviving fraction was measured after each dose level (Figure 5). Regarding the surviving fractions of MDA MB 231 cells, imatinib is able to reduce the surviving fraction further when combined with irradiation. These effects can also be seen in the lower proliferating cell line MCF 7. Here the combined treatment leads to a decrease of the surviving fraction which is explicitly detectable after application of higher Gy levels. Compared to each single treatment, the combined application of irradiation and imatinib further reduces the number of surviving and colony forming cells.

### PDGFR β expression is not effected by irradiation and imatinib

**Figure 6:**
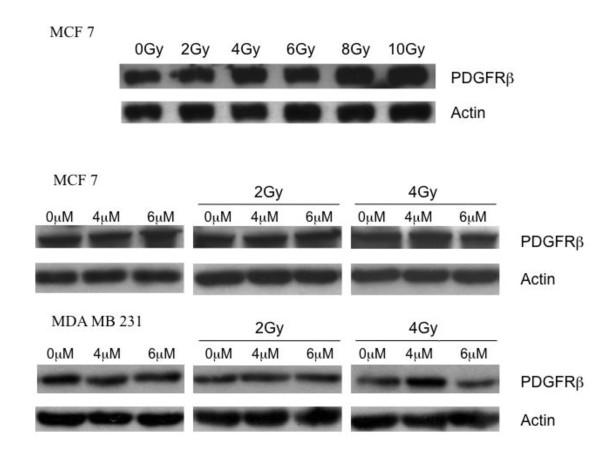
**PDGFR β expression is not effected by irradiation and imatinib**. Breast cancer cell lines underwent irradiation, incubation with imatinib or the combination of both. Total protein was extracted and western blot was carried out using antibodies against PDGFR β.

As the PDGF receptor β is expressed by all cell lines we decided to further investigate the modulation of this receptor by imatinib. To detect if changes in PDGFR β expression occur after imatinib treatment and/or fractionated irradiation, western blot analysis was performed following different treatment modalities. After each treatment option, protein lysates were extracted. As shown in figure 6, the expression of the tyrosine kinase receptor PDGFR β is not modified by the different treatment options in MCF 7 and MDA MB231 breast cancer cell lines.

### Imatinib causes PDGFR β inactivation in combination with irradiation

**Figure 7:**
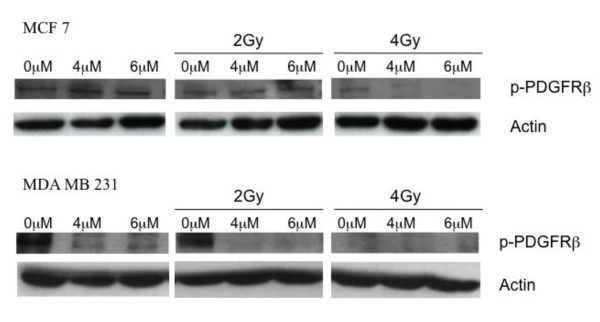
**Imatinib causes PDGFR β inactivation in combination with irradiation**. Cell lines were treated with imatinib alone or in combination with irradiation. Total protein was extracted and western blot analysis was carried out using antibodies against phospho-PDGFR β. Equal loading was confirmed by β-Actin detection.

Modulation and inhibition of the PDGFR β signalling pathway by imatinib can be measured by the receptor phosphorylation status. Tests were carried out after single incubation with imatinib and in combination with irradiation. MCF 7 cells show an inhibition of receptor activation when imatinib in a concentration of 6 μM is combined with irradiation (Figure 7). An effect of single imatinib incubation on receptor phosphorylation could not be detected in this experimental set-up. In contrast to these results imatinib is able to inhibit PDGFR β phosphorylation in MDA MB 231 cells. After incubation with imatinib in concentrations of 4 and 6 μM receptor activation is blocked. In irradiated breast cancer cells this effect on receptor inactivation is consistent.

## Discussion

The aim of this study was to detect which effect the tyrosine kinase inhibitor imatinib mesylate has on breast cancer cell lines in vitro when combined with either chemotherapy or irradiation. Further it was examined how imatinib modulates the PDGFR β signalling pathway. For this purpose, expression patterns of imatinib specific target receptors PDGFR α, β, c-kit and their according ligands PDGF A, B and H-SCF-1 were examined. While PDGFR β was detectable among all cell lines, PDGFR α was not expressed by MCF 7 cells in this setting, but they were positive for c-kit. The ligands PDGF A and B were transcribed by both cell lines and H-SCF-1 only by MCF 7. These expression patterns correspond with other studies which describe an expression of the ligand PDGF A but not the PDGFR α receptor by MCF 7 cells [35]. So far only few groups have performed immunohistochemical analysis in primary breast cancers on PDGFR expression which plays an important role in autocrine and paracrine stimulation of cancer and stromal cells as well as in angiogenesis [13]. An expression of PDGFR α could be detected in 40% of ductal invasive breast cancers and more than 80% of these showed also a PDGF A translation. A positive correlation between receptor expression and axillary lymph node metastasis has been suggested [35]. PDGFR β is expressed in endothelial cells of ductal carcinoma in situ [36] and also of prognostic value in peritumoral stroma [37]. Positivity has been reported for over 50% of invasive carcinoma cells.

Furthermore, autocrine stimulation of PDGF receptors is reported as an important factor in tumor progression and metastasis [38,39]. Likewise poor overall survival rates and low response to chemotherapy are correlated with over-expression of PDGF receptors [23]. Therefore by targeting these receptors, imatinib might be able to inhibit local tumour growth and decrease metastatic potential.

In previous studies we were able to determine a cell growth inhibition of imatinib on breast cancer cell lines in clinically relevant concentrations of 4 to 6 μM which could be confirmed by other groups [31,34]. In other cancer entities such as pancreatic cancer or medullary cancer of the thyroid gland higher concentrations were needed to cause cell growth inhibition in vitro which might be due to different receptor expression patterns [40,41]. Oral application of 25-600 mg imatinib can lead to plasma levels of about 0.17-5.68 μM in vivo [42]. Therefore in vivo effects of imatinib in breast cancer patients appear to be reasonable in a tolerable dose.

To further investigate the interaction of imatinib with growth signalling pathways, we have chosen the PDGFR β axis because this receptor is expressed among both cell lines examined. Moreover it is not sure if c-kit activation mediates cell proliferation in breast cancer [14,43]. At least we were not able to detect an increased cell growth through SCF (c-kit ligand) application in previous experiments, whereas incubation with PDGF BB leads to augmented cell growth in all breast cancer cell lines and has the ability to stimulate cell migration. Similar results could be detected by other research groups who could show that PDGF BB application leads to increased cell growth whereas PDGF AA had no effect on cell proliferation [44]. Moreover, in ovarian and breast cancer cell lines an inhibition of PDGF BB generated PDGFR β phosphorylation could be detected after imatinib incubation [19,27,31].

PDGFR β activation is known to cause a protective effect against the antiproliferative potential of chemotherapeutic drugs as paclitaxel and doxorubicin in various cancer entities [28,45]. For this reason, the effect of a combination of imatinib, as a PDGFR β inhibitor, with chemotherapeutic drugs in breast cancer cells appeared to be promising. In previous experiments we could determine a significant cell growth inhibition when imatinib was combined with vinorelbine compared to a single vinorelbine treatment [31]. Moreover a combination of imatinib with cisplatin has an additive effect on cell growth inhibition [46]. In this experimental setting we studied the effect of imatinib in combination with doxorubicin, which is frequently used in the adjuvant and palliative therapy of breast cancer.

In combination with doxorubicin an increase in chemosensitivity due to the potential of imatinib to inhibit PDGFR β could be demonstrated among feline vaccine-associated sarcomas [47]. In this experimental setting we were able to prove that imatinib, despite its potential of decreasing the interstitial pressure in tumours and enhancing anti-angiogenic effects in vivo, is able to alter the chemosensitivity of breast cancer cell lines in vitro. This is of major interest because clinical trials investigating the effect of imatinib either as a monotherapy or combined with docetaxel in metastasized breast cancer do not show significant results even if patients were selected according to PDGF receptor expression of tumour cells [48-50]. For this reason, it is important to perform further trials combining imatinib with chemotherapeutic drugs. A selection of patients expressing targets for imatinib treatment is of great relevance.

Irradiation is an important component of standard therapy in the adjuvant as well as in the metastasized setting of breast cancer. For this reason, we investigated the effect of irradiation and the combination with imatinib on breast cancer cell lines in vitro. Comparing the two breast cancer cell lines MCF 7 and MDA MB 231, there is some inhomogenity in the anti-proliferative effect caused by ionizing radiation. MDA MB 231 has a higher mitotic potential and therefore a higher plating efficiency compared to MCF 7. Moreover, the surviving fraction of MDA MB 231 compared to MCF 7 cells according to the radiation doses is diminished in the cell colony forming test. The demonstrated differences in radiosensitivity of both breast cancer cell lines correlate with formerly published results for unfractionated irradiation [51,52]. To investigate the effect of imatinib on radiosensitivity of breast cancer cell lines, cells were incubated with imatinib and a fractionated radiation was applied. Comparing the surviving fractions of cells undergoing radiation only or imatinib incubation only with the combination of both demonstrates that the combination treatment leads to a further decrease in cell survival in the two cell lines. The decrease is detectable among all dose levels of radiation. These results are similar to other investigations on radiosensitivity modulation through imatinib in different cancer cell lines in vitro [20,53-55]. The effect of a combination of imatinib and radiation was studied in particular in glioblastomas. Incubation with imatinib increases the radiosensitivity in glioblastoma cell lines whereas there is no detectable alteration among fibroblasts [56-58]. The effectivity of this combinatory treatment can not yet fully be explained. A possible explication might be an inhibition of the PDGF receptor pathway by imatinib, which interacts with the alterations of cell signalling, caused by radiation. By inhibiting the PDGF receptor signalling, there might occur a blockade of cell repair mechanisms which are important in repairing radiation induced damages like double-strand breaks on the DNA level. In PDGFR expressing glioblastoma cells the addition of imatinib to irradiation induced a significant increase of cells undergoing apoptosis. The effect of radiation could be improved through imatinib enhanced apoptosis rates [53].

As well as for chemotherapy, a high interstitial fluid pressure (IFP) is also a prognostic marker for poor response on irradiation [59]. For this reason a diminished IFP due to imatinib mediated PDGFR inhibition could be another mechanism to alter the effects of irradiation. We could show that there is no change in PDGFR β expression when cells were incubated with single imatinib or in combination with radiation. Single irradiation has no effect on receptor activation whereas combination with imatinib leads to an inhibition of receptor phosphorylation in both cell lines. This effect was formerly described in other cancer entities like glioblastomas, ovarian cancer and small cell lung cancer [10,19,60]. According to our results, single radiation does not cause any changes in PDGFR β activity in glioblastoma cells, whereas a direct correlation between imatinib induced inhibition of PDGFR β phosphorylation and increased radiosensitivity was detected [57]. Imatinib is able to modify several different cell signalling pathways and has therefore diverse possibilities in modulating the effects of irradiation. It is certain that inhibition of the PDGFR β pathway plays a decisive role in the enhancement of radiosensitivity in breast cancer cell lines.

## Conclusions

Imatinib as a tyrosine kinase inhibitor has the ability to modulate receptor pathways. In our study we could demonstrate that imatinib is able to inhibit the autocrine and paracrine mediated activation of the PDGFR β pathway and therefore to inhibit the PDGF BB induced proliferation as well as migration of breast cancer cell lines in vitro. In combination with the chemotherapeutic drug doxorubicin, an additive effect on the inhibition of cell proliferation could be detected. If combined with fractionated radiotherapy a decrease in the surviving fraction of breast cancer cells compared to single treatment is caused by imatinib. A possible explanation for this enhanced radio- and chemosensitivity seams to be the imatinib mediated inhibition of the PDGFR β pathway.

Further clinical trials have to be performed to approve these effects on breast cancer in vivo. A strong selection of patients expressing targets for imatinib is required to survey the influence of imatinb in combination with radiation or chemotherapy in breast cancer patients. Overall the results of this study suggest imatinib to be a candidate as an effective add-on in breast cancer related chemo- and radiotherapy. For this reason, imatinib remains a promising drug in the treatment of breast cancer.

## Competing interests

We confirm that all authors fulfil all conditions required for authorship. We also confirm that there is no potential conflict of interest or financial dependence regarding this publication, as described in the Instruction for Authors.

## Authors' contributions

MTW participated in the design of the study, carried out the drug interaction and apoptosis assays, performed the statistical analysis and drafted the manuscript. LD helped carrying out the drug interaction assays. CS participated in the study design. DOB helped drafting the manuscript. KW carried out the immunoblotting and participated in the irradiation. PN carried out the irradiation and was involved in the study design. MB was involved in the data analysis and helped drafting the manuscript. AS helped to draft the manuscript. WJ was involved in the study design. NM helped to draft the manuscript. CM participated in the study design and its coordination and helped to draft the manuscript. All authors have read and approved the manuscript.

## Pre-publication history

The pre-publication history for this paper can be accessed here:

http://www.biomedcentral.com/1471-2407/10/412/prepub
